# FcγR–ACE2 cooperative antibody-dependent enhancement in human and veterinary coronaviruses: mechanistic insights, comparative immunology, and implications for nano-engineered immunomodulatory platforms

**DOI:** 10.3389/fimmu.2026.1859321

**Published:** 2026-07-06

**Authors:** Harishkumar Jeethalu Neelakantan

**Affiliations:** Department of Veterinary Pharmacology and Toxicology, Veterinary College and Research Institute, Tamil Nadu Veterinary and Animal Sciences University (TANUVAS), Thanjavur, Tamil Nadu, India

**Keywords:** ACE2, afucosylation, antibody-dependent enhancement, coronavirus immunopathology, cytokine storm, Fc engineering, Fc gamma receptor, FIPV

## Abstract

Antibody-dependent enhancement (ADE) is a paradoxical immunological phenomenon in which pre-existing antibodies facilitate viral entry into host cells rather than conferring protection. ADE has been extensively characterised in flaviviral systems, most notably dengue virus (DENV), and presents a significant challenge for vaccine development and antibody-based therapeutic design. In coronavirus infections, ADE may operate through both classical Fc gamma receptor (FcγR)-mediated pathways and an intrinsic signalling mechanism involving inhibitory FcγRIIb-mediated suppression of the type I interferon (IFN-I) response. Of critical translational relevance is the functionally demonstrated cooperative FcγR–ACE2 entry model for SARS-CoV-2, wherein virus–antibody immune complexes engage Fcγ receptors and require ACE2 interaction for efficient enhancement. For SARS-CoV-2 specifically, ADE magnitude appears to be determined by an antibody’s capacity to block spike–ACE2 interaction rather than its neutralisation potency *in vitro*—a finding distinct from FIPV and other coronavirus ADE systems where classical FcγR-mediated mechanisms predominate without ACE2 co-receptor dependency. Feline infectious peritonitis virus (FIPV) represents one of the most rigorously documented biological systems in which antibody-mediated macrophage infection directly determines systemic disease outcome. This comprehensive review integrates current knowledge of FcγR biology, IgG subclass dynamics, antibody glycosylation, coronavirus cell entry mechanisms, intracellular signalling cascades, cytokine dysregulation, comparative veterinary immunopathology, and nano-engineered immunomodulatory platforms for ADE-safe vaccine development. No confirmed clinical ADE has been documented to date in mRNA-vaccinated populations, though theoretical risk windows and population-specific vulnerabilities are critically discussed.

## 1 Introduction

The global emergence of SARS-CoV-2 and the COVID-19 pandemic renewed scientific scrutiny of antibody-dependent enhancement (ADE), a process in which virus-specific antibodies paradoxically facilitate rather than prevent cellular infection (1, 2). ADE was first described in relation to dengue virus (DENV) in the 1960s and has since been reported in HIV, Zika, West Nile, and multiple coronaviruses (1, 3).

Ebola ADE has been reported in pseudovirus and cell-culture systems (3), though its *in vivo* clinical significance is less well-validated compared to dengue haemorrhagic fever and should not be equated in evidentiary weight. The classical ADE model involves Fc gamma receptor (FcγR)-mediated endocytosis of IgG-opsonised virions into immune cells (2–4). A second pathway—intrinsic ADE—operates via inhibitory FcγRIIb signalling, suppressing antiviral interferon production without necessarily altering viral tropism (4, 5).

The mechanistic complexity of ADE in coronaviruses is compounded by the unique biology of these pathogens (6, 7). Unlike flaviviruses, coronaviruses primarily infect epithelial cells through spike glycoprotein interaction with specific host receptors: ACE2 for SARS-CoV-2 and SARS-CoV-1; aminopeptidase N (APN/CD13) for HCoV-229E and feline coronavirus; and DPP4 for MERS-CoV (6, 7). Wang et al. (8) specifically demonstrated that ACE2 can function as a secondary receptor in FcγRdependent ADE of SARS-CoV-2 infection, providing foundational evidence for the cooperative entry model central to this review. Kuzmina et al. (9) further validated this cooperative mechanism using a panel of 364 CoVIC IgG1 mAbs. These findings derive primarily from *in vitro* THP-1 cell studies and their extrapolation to *in vivo* clinical contexts requires further validation (8, 9). Recent structural advances using cryo-electron tomography (cryo-ET) have begun to illuminate the molecular geometry of spike-antibodyreceptor interactions (10, 11), opening new avenues for understanding cooperative receptor engagement in ADE.

## 2 Molecular basis of antibody-dependent enhancement

### 2.1 Classical extrinsic ADE: FcγR-mediated viral entry

**Figure 1:**
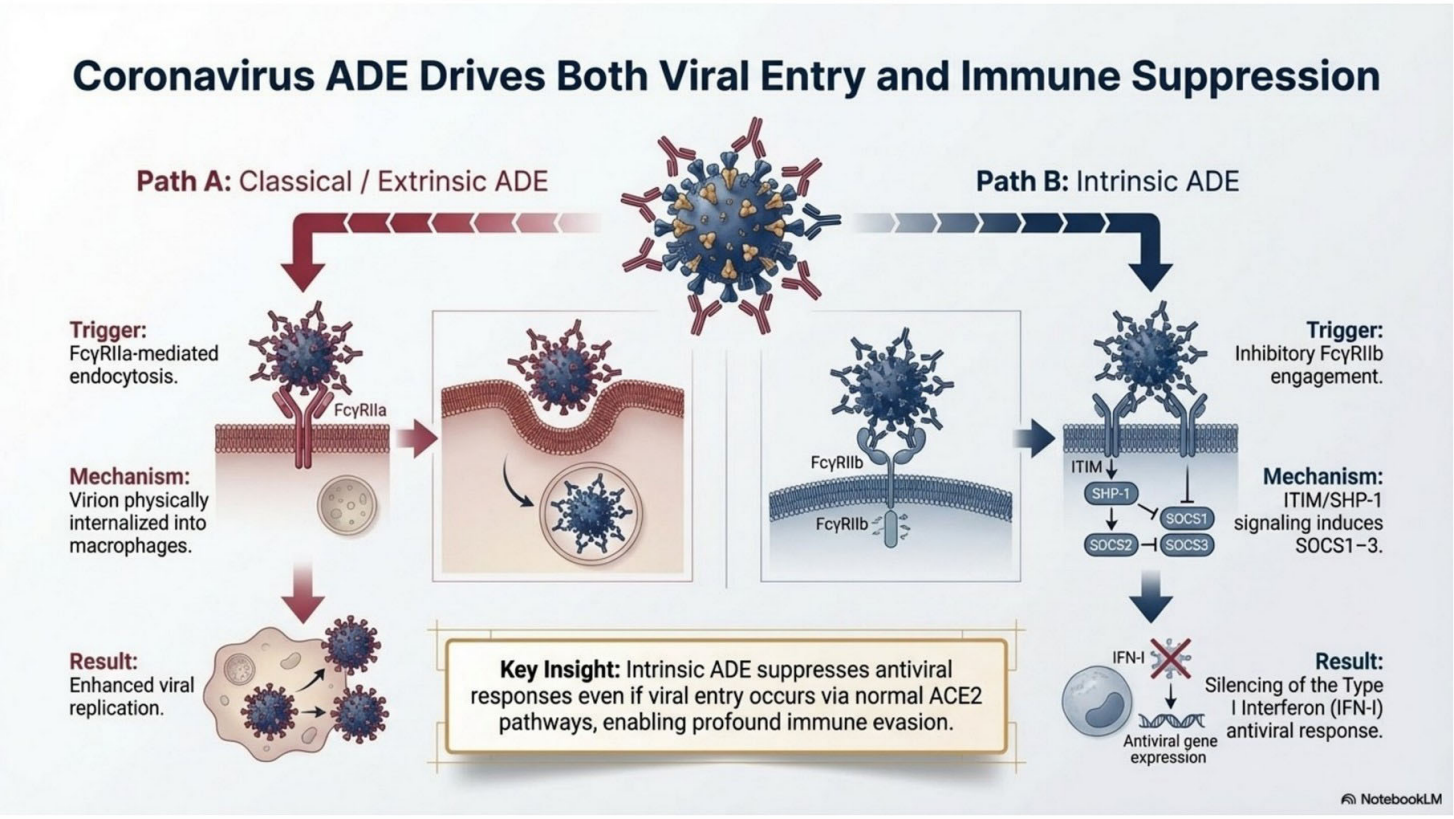
Two mechanistic pathways of antibody-dependent enhancement (ADE) in coronavirus infections (4, 5, 12, 13). Path A (Classical/Extrinsic ADE): Sub-neutralising IgG enables FcγRIIa-mediated endocytosis of virus– antibody immune complexes into macrophages, resulting in enhanced viral replication and cytokine storm. Path B (Intrinsic ADE): Inhibitory FcγRIIb engagement activates ITIM/SHP-1–2/SOCS1–3 signalling, silencing the IFN-I antiviral response and enabling profound immune evasion without necessarily altering cellular tropism. Key Insight: Intrinsic ADE suppresses antiviral responses even if viral entry occurs via normal ACE2 pathways.

Classical extrinsic ADE involves the direct facilitation of viral entry into immune cells via Fc receptormediated internalisation of antibody-opsonised virions (1, 2). When viral neutralisation is incomplete—at sub-neutralising antibody concentrations, with low-affinity antibodies, or against heterologous antigenic variants—virus–antibody immune complexes trigger clathrin-mediated endocytosis, delivering replicationcompetent virions to intracellular compartments (**Figure 1**) (12, 13). Kibria et al. (12) demonstrated antibody-mediated SARS-CoV-2 entry in THP-1 cells. Wieczorek et al. (13) established that neutralisation potency is a poor predictor of ADE magnitude *in vitro*—consistent with the cooperative receptor model (8, 9).

### 2.2 Intrinsic ADE: FcγRIIb-mediated IFN-I suppression

Beyond classical extrinsic ADE, coronaviruses may exploit an intrinsic ADE pathway mediated through the inhibitory receptor FcγRIIb (CD32b) (4, 5). FcγRIIb signals through an immunoreceptor tyrosine-based inhibitory motif (ITIM), recruiting phosphatases SHP-1 and SHP-2 (14, 15). Engagement of FcγRIIb by IgG-opsonised SARS-CoV-2 converges on the JAK-STAT pathway to suppress IFN-I production via induction of SOCS1 and SOCS3 (4, 5). Matveeva (16) provided evidence that SARS-CoV-2 infection of phagocytic immune cells proceeds via FcγR-dependent pathways with downstream immunosuppressive consequences including IFN-I blunting. Intrinsic ADE can operate independently of altered viral entry route —IFN-I suppression affects the entire antiviral transcriptional programme regardless of viral entry mechanism (4, 5).

### 2.3 Antibody glycosylation and ADE risk

The glycosylation state of the IgG Fc region at Asn297 profoundly influences FcγR affinity and ADE risk (17, 18). Afucosylated IgG1 exhibits dramatically enhanced binding to FcγRIIIa, increasing ADCC and theoretical ADE potential (17). Elevated afucosylated anti-spike IgG has been reported in severe COVID-19 patients following natural infection (17). Regarding mRNA vaccination, while sustained afucosylation is largely absent in antigen-experienced vaccinees, Van Coillie et al. (19) demonstrated that BNT162b2 induces a transient afucosylated anti-spike IgG1 response in naive individuals during the first 2–6 weeks post-primary dose. This early temporal window theoretically represents a period of transiently elevated FcγRIIIa-mediated ADE risk, though no clinical ADE has been reported in this period (19).

### 2.4 IgG subclass dynamics and ADE risk: the IgG4 dimension

Repeated SARS-CoV-2 mRNA vaccination induces a progressive class switch toward spike-specific IgG4 antibodies, rising significantly after the second and third doses (20, 21). IgG4 exhibits reduced binding to activating FcγRs (FcγRI, FcγRIIa, FcγRIIIa) while maintaining affinity for the inhibitory FcγRIIb (22), suggesting a paradoxical profile: reduced classical ADE risk but potentially compromised neutralising capacity (20, 21). Irrgang et al. (20) demonstrated this class switch is associated with reduced antibodydependent phagocytosis and increased risk of breakthrough infections (23). Uversky et al. (21) proposed IgG4 induction may generate immune tolerance to spike protein. The IgG4 shift represents a nuanced consideration for ADE-safe vaccine design requiring ongoing longitudinal surveillance (20–22).

## 3 Fc gamma receptor biology in immune cells

### 3.1 Classification, structure, and expression

Fc gamma receptors are encoded on chromosome 1q23, comprising six distinct proteins: FcγRI (CD64), FcγRIIa (CD32a), FcγRIIb (CD32b), FcγRIIc (CD32c), FcγRIIIa (CD16a), and FcγRIIIb (CD16b) (14, 15). Binding affinities range from ~10^9^ M for FcγRI to ~10^5^ M for FcγRIIIb (14, 15) (**Table 1**). GM allotypes^−−^ and FcγR genotypes interact to contribute to ADCC magnitude against SARS-CoV-2 (24).

**Table 1:** Classification and properties of human Fc gamma receptors relevant to ADE (14, 15).

FcγR	CD	Kd	Expression	Motif	ADE relevance
FcγRI	CD64	~10^9^ M^−^	Monocytes, Macrophages, DCs	ITAM	Primary ADE mediator; binds monomeric IgG; blockade most pronounced in ADE (9, 14)
FcγRIIa	CD32a	~10^7^ M^−^	Macrophages, Neutrophils, Platelets	ITAM	Activating; central coronavirus ADE receptor; H131R polymorphism (14, 15)
FcγRIIb	CD32b	~10^7^ M^−^	B cells, Macrophages, DCs	ITIM	Sole inhibitory FcγR; intrinsic ADE; IFN-I suppression via SOCS; IgG4 preferential (4, 5, 22)
FcγRIIIa	CD16a	~10^6^ M^−^	NK cells, Macrophages, Monocytes	ITAM	ADCC; high-avidity ADE; V158F polymorphism; enhanced by afucosylated IgG (14, 17, 18)
FcγRIIIb	CD16b	~10^5^ M^−^	Neutrophils only	GPI	Phagocytosis; minimal direct ADE role (14)

ITAM, immunoreceptor tyrosine-based activation motif; ITIM, inhibitory motif; Kd, dissociation constant; DCs, dendritic cells.

### 3.2 Intracellular signalling cascades in ADE

**Figure 2:**
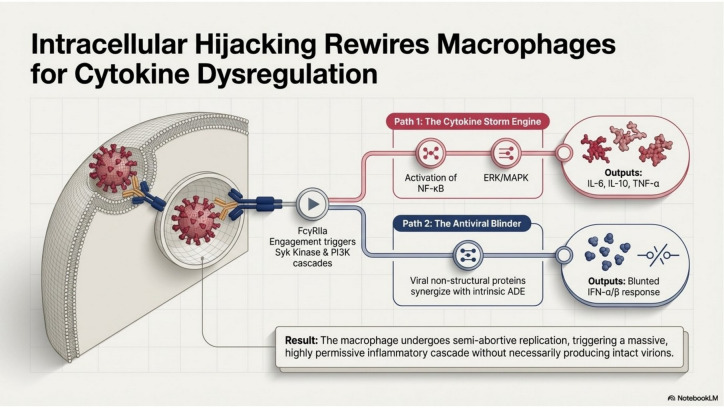
Intracellular signalling cascade downstream of FcγRIIa engagement during ADE (4, 5, 14, 15). FcγRIIa engagement triggers Syk kinase and PI3K activation, diverging into two pathogenic outputs: Path 1 (Cytokine Storm Engine) activating NF-κB/ERK-MAPK, producing IL-6, IL-10, and TNF-α; Path 2 (Antiviral Blinder) converging with viral non-structural proteins to synergistically blunt the IFN-α/β response. The macrophage undergoes semi-abortive replication, triggering a massive, highly permissive inflammatory cascade without necessarily producing intact virions (16).

Activating FcγR signalling in macrophages proceeds through Src-family kinase phosphorylation of ITAM tyrosines, generating docking sites for Syk kinase (14, 15). Syk activates PLCγ → IP3/DAG, mobilising intracellular calcium and PKC. Parallel PI3K → PIP3 → Akt/mTOR activation, together with NF-κB and ERK/MAPK transcriptional activation, produces cytokine secretion and phagocytosis (14, 15). In ADE, this cascade is co-opted by internalised virions, producing a dual outcome: enhanced IL-6/IL-10/TNF-α production via the cytokine storm pathway, and blunted IFN-α/β response via viral non-structural protein synergy with intrinsic ADE (**Figure 2**) (4, 5, 16, 25, 26).

### 3.3 FcγR polymorphisms and individual ADE susceptibility

The FcγRIIa-H131R polymorphism has been associated with differential dengue haemorrhagic fever susceptibility through *in vitro* studies and epidemiological observations (14). H131 homozygotes bind IgG immune complexes with higher affinity, driving highly efficient FcγR-mediated viral uptake. This polymorphism is hypothesised to influence macrophage ADE risk in SARS-CoV-2 infection, though no prospective clinical study has yet validated FcγR polymorphisms as predictive biomarkers for COVID-19 ADE (14, 24). GM allotypes and FcγR genotypes have been demonstrated to interact in contributing to ADCC magnitude against SARS-CoV-2 spike-expressing cells (24).

## 4 Coronavirus cell entry mechanisms

### 4.1 Spike glycoprotein architecture and RBD–ACE2 interaction

The coronavirus spike (S) glycoprotein is a class I fusion protein existing as a homotrimer (6, 7). S1 contains the receptor-binding domain (RBD) and N-terminal domain (NTD); S2 mediates membrane fusion. SARS-CoV-2 RBD engages ACE2 through a buried surface area of ~864 Å^2^, involving key contact residues Lys417, Leu452, Tyr453, Gln493, Ser494, Asn501, and Tyr505 (6, 7). A multibasic furin cleavage site (PRRAR↓S) at the S1/S2 boundary enables pre-activation of virions during biogenesis, facilitating direct TMPRSS2-mediated fusion (6). Recent cryo-ET studies have provided detailed structural characterisation of spike-mediated membrane fusion intermediates (10, 11), offering structural foundations relevant to cooperative receptor engagement during ADE.

### 4.2 Dual entry routes: TMPRSS2-mediated fusion vs endosomal cathepsin pathway

SARS-CoV-2 employs two principal entry routes: (1) plasma membrane fusion dependent on TMPRSS2, and (2) endosomal entry via cathepsins B/L following clathrin-mediated endocytosis (6, 27). The balance between these routes is critically altered in ADE scenarios where FcγR-mediated endocytosis directs virions into the endosomal compartment regardless of TMPRSS2 availability (12, 13). The Omicron variant spike– ACE2 complex structure (26) demonstrates how variant-specific structural changes influence receptor binding and the cooperative ADE model’s applicability across emerging variants.

## 5 The cooperative FcγR–ACE2 entry model

### 5.1 Rationale, structural feasibility, and experimental evidence

**Figure 3:**
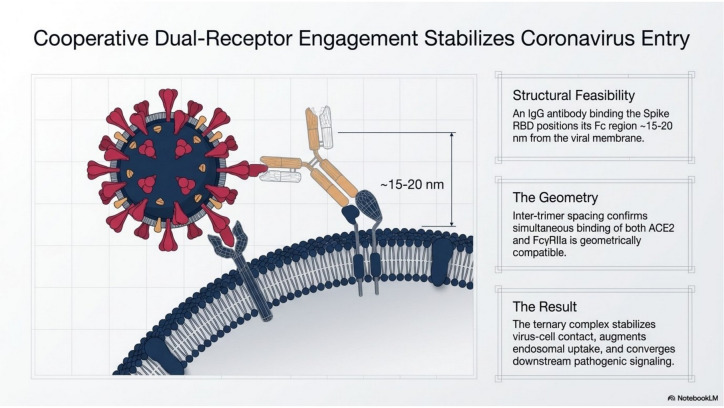
Structural feasibility of the cooperative FcγR–ACE2 dual-receptor entry mechanism in SARS-CoV-2 (8, 9, 11). An IgG antibody binding the spike RBD positions its Fc region ~15–20 nm from the viral membrane— structurally compatible with simultaneous engagement of both FcγR and ACE2 on the macrophage surface. The ternary virus–antibody–cell complex stabilises virus–cell contact, augments endosomal uptake, and converges downstream pathogenic signalling. The immunological consequence: neutralisation potency (IC50) shows no significant correlation with ADE magnitude (R^2^ = 0.0098) *in vitro*, demonstrating that antibodies blocking ACE2 binding are paradoxically high-ADE-risk.

The FcγR–ACE2 cooperative model, as recently elucidated by Kuzmina et al. (9) and mechanistically supported by Wang et al. (8), indicates that ADE in SARS-CoV-2 is not mediated by FcγR engagement alone but is facilitated by cooperative interaction with ACE2 as a co-factor. Wang et al. (8) specifically demonstrated that ACE2 can function as a secondary receptor in FcγR-dependent ADE of SARS-CoV-2 infection. These findings derive primarily from *in vitro* THP-1 cell studies and their extrapolation to *in vivo* clinical scenarios requires further validation (8, 9, 28). As shown in **Figure 3**, an IgG antibody binding the spike RBD positions its Fc region approximately 15–20 nm from the viral membrane—a geometry compatible with simultaneous FcγR and ACE2 engagement (9, 11).

Key experimental evidence from Kuzmina et al. (9) includes: (i) 364 CoVIC IgG1 mAbs screened against THP-1 cells demonstrating dose-dependent ADE peaking at 1 μg/mL, with neutralisation potency (IC50) showing no significant correlation with ADE magnitude (R^2^ = 0.0098) in this *in vitro* system; (ii) ADE was completely abrogated by combined FcγR and ACE2 blockade; (iii) mAbs with high ACE2-blocking capability (>50% RBD–ACE2 inhibition) were disproportionately associated with strong ADE; and (iv) ADE-mediated infection was productive in THP-1 cells (9). Crucially, ceftazidime used in these experiments acts as a spike RBD-directed interface disruptor that sterically hinders spike–ACE2 interaction by binding the spike RBD (29)—not an ACE2 receptor antagonist. Other groups have reported abortive or semi-productive infection in primary macrophages via ADE, suggesting THP-1 findings may not fully recapitulate primary macrophage biology (16).

Nakayama and Shioda (37) and Tao et al. (38) have provided comprehensive reviews of SARS-CoV-2related ADE phenomena both *in vitro* and *in vivo*, reinforcing the mechanistic coherence of the cooperative entry model while emphasising the need for additional *in vivo* validation and the absence of confirmed clinical ADE in mRNA-vaccinated populations.

### 5.2 Implications for antibody-based therapeutics

The cooperative entry model has direct implications for therapeutic monoclonal antibody (mAb) design (8, 9, 14, 30, 31). LALA mutations (L234A/L235A) and LALAPG mutations abrogate activating FcγR binding while preserving Fab functionality (14, 31). The GASDALIE mutation set (G236A/S239D/A330L/I332E) enhances FcγRIIIa engagement for therapeutic ADCC applications (30, 31). YTE mutations (M252Y/S254T/T256E) extend antibody half-life while modulating FcγR interactions (14, 31). Hale (31) provides a comprehensive review of 40 years of Fc engineering strategies covering LALA, LALAPG, GASDALIE, and related variants, confirming that rational Fc engineering can selectively modulate ADE risk without compromising therapeutic efficacy.

## 6 Intracellular trafficking and viral fate after FcγR-mediated entry

Following FcγR-mediated endocytosis, virus-containing phagosomes undergo progressive acidification through early endosomes (EEA1, Rab5) to late endosomes (LAMP-1, Rab7) (12, 16). *In situ* cryo-ET^++++^ by Akıl et al. (10) revealed that SARS-CoV-2 spike undergoes extensive structural rearrangements through fusion intermediates prior to fusion-pore formation, providing mechanistic context for how FcγR-mediated endosomal redirection may alter the fusion process compared to ACE2-mediated plasma membrane fusion. Within macrophages infected via ADE, SARS-CoV-2 replication may proceed through the replicationtranscription complex (RTC) (16). Importantly, macrophage-infected coronaviruses frequently undergo abortive or semi-abortive replication—sufficient for viral RNA synthesis and protein production but without generating abundant infectious progeny (16). SARS-CoV-2 encodes at least 16 non-structural proteins and multiple accessory proteins that antagonise IFN-I production at multiple points: RIG-I/MDA5 inhibition via nsp3-PLpro, STING-TBK1-IRF3 signalling disruption via nsp13 ATPase, STAT1/2 nuclear translocation inhibition via ORF6, and ISG expression blockade via nsp1 ribosome stalling (4, 25). Combined with FcγRIIb-SOCS-mediated extrinsic IFN-I suppression, these mechanisms may synergise to create a profoundly immunosuppressive intracellular environment (4, 16, 25).

## 7 Veterinary coronavirus ADE: the FIPV paradigm

### 7.1 FCoV biology and the biotype conversion to FIPV

**Figure 4:**
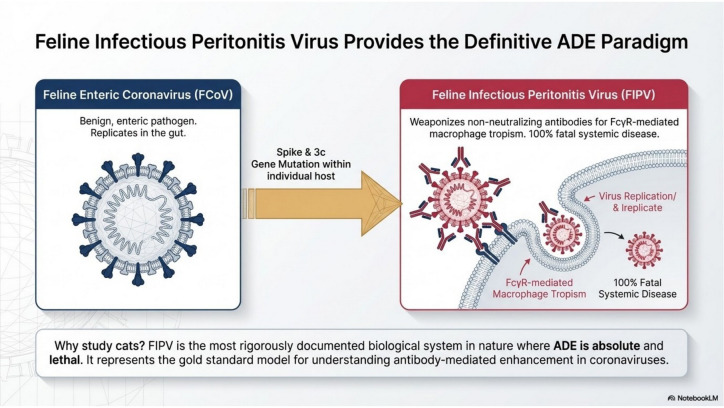
Feline Infectious Peritonitis Virus (FIPV) provides the definitive ADE paradigm (32–35). Spike and 3c gene mutation within an individual host converts the benign feline enteric coronavirus (FCoV) into FIPV, which weaponises non-neutralising anti-FCoV IgG for FcγR-mediated macrophage tropism. The FcγR functions here as an immune-complex uptake receptor—not a direct viral entry receptor—enabling virus replication and dissemination via infected macrophages to produce pyogranulomatous vasculitis and.severe systemic disease historically associated with high mortality in untreated cats FIPV is among the most rigorously documented biological system demonstrating a central role for ADE in coronavirus pathogenesis.

Feline coronavirus (FCoV) is a prevalent enteric pathogen of domestic and wild felids, with seroprevalence reaching 90% in multi-cat environments (32, 33). Two biotypes are recognised: feline enteric coronavirus (FECV)—a benign enteric pathogen—and feline infectious peritonitis virus (FIPV), the causative agent of a historically highly fatal systemic disease FIP (32, 33). The FECV-to-FIPV biotype conversion (independent of Type I/II serotype classification) arises through spontaneous mutations primarily in the spike S gene and the 3c gene within an individual host (32, 33). The critical distinction is biological: FIPV weaponises nonneutralising antibodies for FcγR-mediated macrophage tropism, producing severe systemic disease historically associated with high mortality in untreated cats (**Figure 4**) (32, 34, 35).

### 7.2 FcγR-mediated macrophage infection: core ADE mechanism

The hallmark virological feature distinguishing FIPV from FECV is pronounced macrophage tropism mediated through antibody-dependent FcγR endocytosis (34, 35). Importantly, FIPV acquires enhanced macrophage tropism through antibody-mediated immune complex endocytosis, where FcγR functions as an immune-complex uptake receptor—not a direct viral entry receptor. FIPV infection of macrophages does not involve direct spike–FcγR binding (34, 35). FcγR blockade with anti-FcγR antibodies completely abrogates FIPV macrophage entry, confirming FcγR-dependent ADE as the obligate macrophage infection pathway under antibody-opsonised conditions (34, 35).

### 7.3 Vaccine-enhanced disease in FIPV: translational lessons

Early FIPV vaccines paradoxically accelerated disease in vaccinated animals—a phenomenon termed ‘early death syndrome’ (AMEFI) (33, 35). Vaccinated cats with non-neutralising anti-spike IgG developed faster, more severe FIP than unvaccinated controls (35). This underscores a critical principle directly applicable to SARS-CoV-2 vaccine design: sub-protective antibody titres may be worse than no humoral response (16, 35) (**Table 2**). The approved intranasal FIPV vaccine (Primucell-FIP, Zoetis) achieves protection through mucosal IgA and T cell responses with minimal systemic IgG that could mediate ADE (32, 33). The recent clinical success of GS-441524 in treating FIP with >80% remission rates validates the viral RdRp as a therapeutic target across alphacoronavirus and betacoronavirus systems (36).

**Table 2:** Comparative analysis of ADE in SARS-CoV-2 vs FIPV (8, 9, 16, 32–36).

Parameter	SARS-CoV-2 (human)	FIPV (feline)
Coronavirus lineage	Betacoronavirus (lineage B)	Alphacoronavirus (FCoV type I/II)
Primary entry receptor	ACE2 (+ TMPRSS2 co-factor) (6, 7)	APN/CD13; antibody-bridged FcγR endocytosis in ADE (34, 35)
ADE mechanism	Extrinsic (FcγRI+FcγRIIa+ACE2 cooperative) + Intrinsic (FcγRIIb-SOCS) (8, 9)	Classical antibody-bridged FcγR immune-complex endocytosis; FcγR is uptake receptor not entry receptor (34, 35)
Key cytokines	IL-6, IL-10, TNF-α; IFN-I suppressed (4, 5, 25)	IL-6, TNF-α, IL-1β; IL-12 reduced (32, 33)
Vaccine risk	No confirmed clinical ADE with mRNA vaccines to date (37, 38)	Documented AMEFI (early death syndrome) with whole-virus and recombinant spike vaccines (33, 35)
Therapeutic antiviral	Remdesivir, Paxlovid, molnupiravir	GS-441524 (remdesivir parent compound) (36)
Natural ADE model	Human — ethical constraints limit controlled challenge	YES — natural cat model; controlled challenge studies feasible (32–35)

Direct comparison of ADE-relevant parameters between SARS-CoV-2 and FIPV.

## 8 Nano-engineered immunomodulatory platforms for ADE-safe vaccination

### 8.1 Antigen engineering principles to minimise ADE risk

The central immunological challenge for ADE-safe coronavirus vaccines is selectively eliciting antibodies against the ACE2-binding interface of the RBD while avoiding non-neutralising IgG against NTD, S2, and non-conserved spike regions (9, 16, 39, 40). The immunological imperative is to elicit neutralising antibodies only against the ACE2-binding interface, depriving the immune system of the targets that typically generate ADE-bridging non-neutralising IgGs. Prefusion-stabilised spike trimers (HexaPro; 2P variant with K986P/V987P; GSAS furin site mutation) maintain the RBD in an immunologically accessible ‘up’ conformation (39, 40). RBD-only constructs eliminate the NTD and S2 domains from the immunogen entirely (39–41).

### 8.2 Lipid nanoparticle mRNA vaccines

LNP-mRNA vaccines represent the most clinically validated nano-engineered coronavirus platform (42, 43). BNT162b2 and mRNA-1273 use ionisable lipid nanoparticles (~80–200 nm) to deliver modified mRNA encoding prefusion-stabilised spike (42, 43). The glycosylation advantage is critical: LNP-mRNA generates predominantly core-fucosylated IgG1 (a safer Fc glycoform) in antigen-experienced vaccinees, avoiding the dangerous afucosylated IgG seen in natural infection, with the exception of a transient afucosylation window in naive vaccinees (19). Transient, localised spike expression avoids prolonged subneutralising antibody windows (16, 42, 43). No confirmed clinical ADE has been documented to date across hundreds of millions of LNP-mRNA vaccine recipients (42, 43).

### 8.3 Nano-vaccine platform comparison

**Figure 5:**
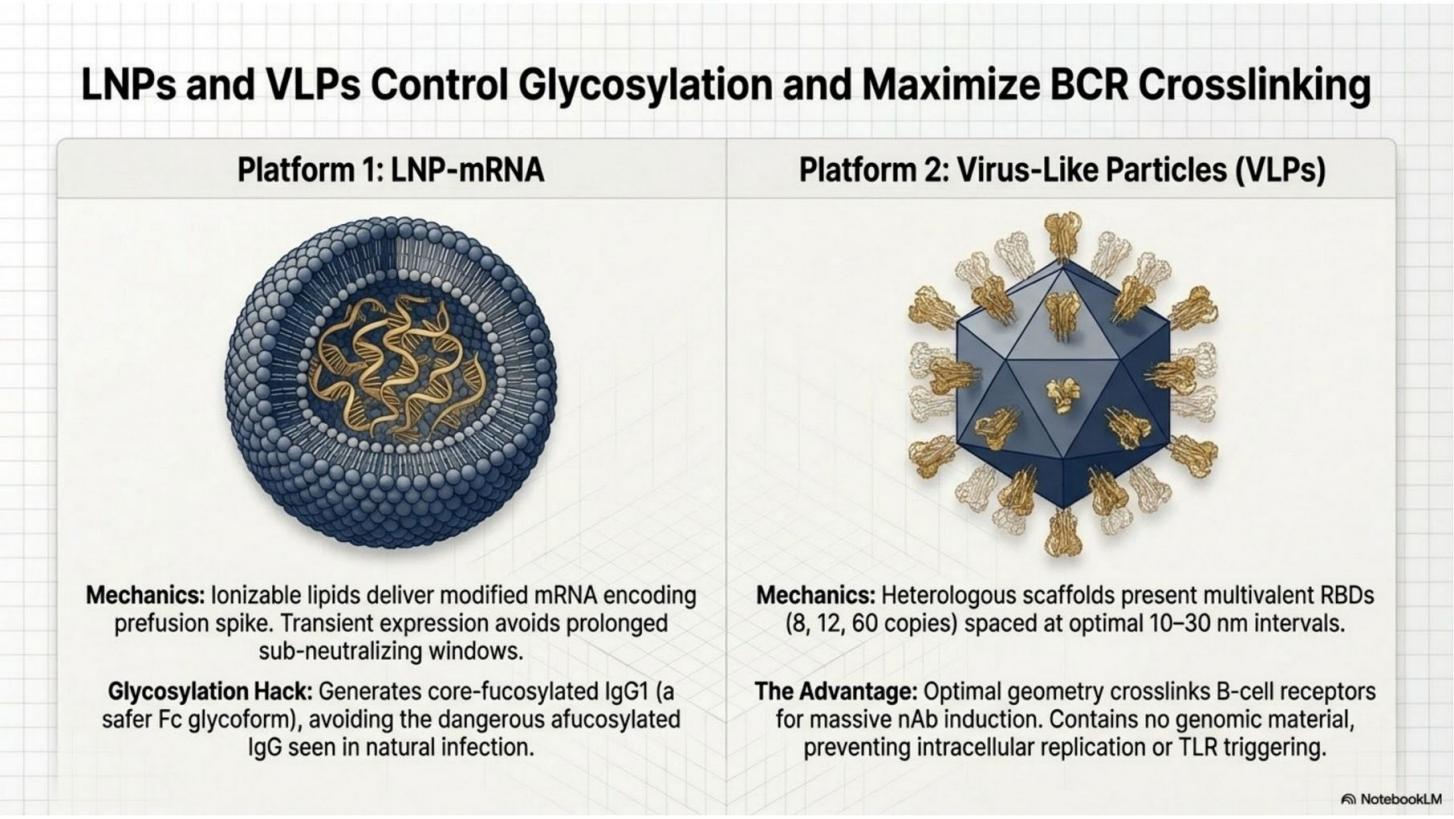
LNPs and VLPs control glycosylation and maximise BCR crosslinking for ADE-safe coronavirus immunisation (16, 39–43). Platform 1 (LNP-mRNA): Ionisable lipids deliver modified mRNA encoding prefusion spike. Transient expression avoids prolonged sub-neutralising windows. Generates core-fucosylated IgG1 (safer Fc glycoform), avoiding the dangerous afucosylated IgG seen in natural infection (19). Platform 2 (Virus-Like Particles): Heterologous scaffolds present multivalent RBDs (8, 12, or 60 copies) spaced at optimal 10–30 nm intervals. Optimal geometry crosslinks B-cell receptors for massive nAb induction. Contains no genomic material, preventing intracellular replication or TLR triggering. The core principle: elicit neutralising antibodies only against the ACE2-binding interface of the RBD.

As illustrated in **Figure 5**, three principal nano-engineered platforms offer distinct ADE-risk mitigation profiles: LNP-mRNA (generating core-fucosylated IgG1, transient antigen expression), virus-like particles (VLPs; heterologous scaffolds presenting multivalent RBDs at optimal 10–30 nm intervals for BCR crosslinking, containing no genomic material), and PLGA/chitosan polymeric nanoparticles (mucosal sIgA induction that does not engage activating FcγRs, biodegradable sustained antigen release) (**Table 3**) (39–41). Since VLPs contain no Fc-activating material and present only the ACE2-binding face of the RBD, they substantially reduce stimulation of antibody lineages associated with ADE risk (9, 39–41).

**Table 3:** Comparative nano-engineered vaccine platform evaluation for ADE-safe coronavirus vaccination (39–43).

Platform	Antigen type	Size	Adjuva nt?	ADE risk mitigation	Stage/examples
LNP-mRNA (42, 43)	Encoded prefusion spike	80–200 nm	No (selfadj)	Core-fucosylated IgG Fc; brief Ag expression; no confirmed clinical ADE to date (37, 38)	Approved: BNT162b2, mRNA-1273
Virus-Like	RBD/spike	20–100	Often	No Fc-activating material;	HPV/HepB approved;
Particle (39, 40)	multimer on scaffold	nm	coformulat ed	multivalent nAb induction; no genomic material	CoV pre-clinical
PLGA NP (40, 41)	Protein/peptide subunit	100–500 nm	MPL, CpG	Sustained release prevents sub-nAb surge window; Th1 bias reduces ADE-prone non-nAb IgG	Pre-clinical CoV; FDAapproved platforms
Chitosan NP (39, 40)	Mucosal protein Ag	100–600 nm	Intrinsic	Mucosal sIgA does NOT engage activating FcγR; substantially reduces classical ADE risk; systemic IgG still possible	Pre-clinical; intranasal route
Self-assembling NP (39, 41)	Ferritin-RBD, I3–01 scaffold	10–50 nm	Alum/AS01B	Precise epitope control; enriches ACE2-blocking nAb lineages; avoids NTD immunodominance	Pre-clinical (HexaPro, SpFN, I53-50)

nAb, neutralising antibody; LNP, lipid nanoparticle; PLGA, poly(lactic-co-glycolic acid).

### 8.4 Biodegradable polymeric nanoparticles (PLGA/chitosan)

PLGA nanoparticles (100–500 nm) provide biodegradable antigen encapsulation with tunable hydrolytic degradation kinetics for sustained antigen release (39–41). Chitosan nanoparticles (100–600 nm) offer mucosal delivery advantages, facilitating induction of secretory IgA (sIgA) (39, 40). sIgA does not engage activating FcγRs on macrophages, substantially reducing classical ADE risk. However, intranasal delivery may still elicit systemic IgG responses in some individuals, and complement-mediated enhancement pathways are not fully excluded (39–41).

## 9 Challenges and controversies in coronavirus ADE

### 9.1 Clinical ADE in COVID-19: evidence assessment

The question of ADE clinical significance in COVID-19 has been contentious (37, 38, 44, 45). Evidence supporting potential ADE relevance includes: (i) *in vitro* demonstrations of antibody-facilitated SARSCoV-2 infection of FcγR-expressing cells (8, 9, 12, 13) (**Table 4**); (ii) correlation of non-neutralising anti-NTD/S2 antibody titres with severe disease (25); (iii) macaque model data suggesting IgG-mediated exacerbation of lung pathology (46); and (iv) the FIPV precedent (34, 35). Counter-arguments include: (i) no confirmed clinical ADE has been documented to date across hundreds of millions of mRNA vaccine recipients (42, 43, 47, 48); (ii) correlation between high neutralising antibody titres and protection is robust (16); (iii) most *in vitro* ADE demonstrations employ non-physiological antibody concentrations or non-primary macrophage lines (12, 13); and (iv) the relative contribution of CTL- and NK-cell-mediated mechanisms to limiting vs amplifying ADE-driven immunopathology *in vivo* remains incompletely characterised. The broader perspectives on ADE risk in the context of SARS-CoV-2 vaccines and therapies have been comprehensively reviewed (47–50). Nakayama and Shioda (37) and Tao et al. (38) provide balanced assessments concluding that mRNA vaccines have successfully avoided clinically significant ADE while emphasising continued surveillance.

**Table 4:** ADE across major pathogenic viruses: mechanisms, target cells, and clinical significance (1–3, 8, 9, 16, 34, 35).

Virus	Family	ADE mechanism	Target cell	Key receptor(s)	Clinical impact
DENV (1, 2)	Flaviviridae	Classical FcγR	Monocytes/Macrophages	FcγRIIa	DHF/DSS; ~25, 000 deaths/yr; strongest clinical ADE evidence
SARSCoV-2 (8, 9, 12, 13)	Coronavirida e	Classical + Intrinsic (cooperative FcγR– ACE2)	Alveolar Macrophages, Monocytes	FcγRI+FcγRI Ia+ACE2	COVID-19 cytokine storm; >6M deaths; cooperative ADE validated *in vitro*
SARSCoV-1 (4, 5)	Coronavirida e	Classical FcγR + Intrinsic	Macrophages, DCs	FcγRI, FcγRII	*In vitro* ADE; vaccine-enhanced disease in animal models
FIPV (34, 35)	Coronavirida e	Classical FcγR (antibodybridged uptake)	Peritoneal Macrophages	Feline FcγR (uptake receptor)	100% fatal FIP; ADE is central, well-characterised; natural model
Zika (1, 3)	Flaviviridae	Classical FcγR	Placental macrophages, DCs	FcγRIIIa	Congenital Zika risk; cross-reactive DENV Ab implicated
Ebola (3)	Filoviridae	Putative FcγR/C1q	Macrophages (*in vitro* only)	FcγR/C1q (*in vitro*)	Pseudovirus/cell-culture only; *in vivo* clinical significance not validated

DHF, dengue haemorrhagic fever; DSS, dengue shock syndrome; FIP, feline infectious peritonitis; DCs, dendritic cells.

### 9.2 Waning immunity, variants, and the ADE threshold

Scenarios where protective antibody titres wane below the neutralisation threshold against antigenically drifted variants represent a nuanced ongoing concern (37, 38, 44, 45). The emergence of Omicron subvariants resulted in significant reductions in neutralisation titres from ancestral-strain vaccination (26). The transient afucosylation window in naive vaccinees (19) and the progressive IgG4 class switch after repeated mRNA vaccination (20, 21) both represent immunological dynamics that theoretically intersect with ADE susceptibility and warrant systematic clinical investigation.

## 10 Future research priorities

- Cryo-electron tomography (cryo-ET): Ternary complex imaging of SARS-CoV-2–IgG–macrophage complexes to directly visualise cooperative FcγR–ACE2 engagement geometry, building on recent *in situ* cryo-ET advances (10, 11).
- Single-cell transcriptomics: sc-RNA-seq/ATAC-seq profiling of macrophages infected via ADE vs standard entry to map ADE-specific transcriptional signatures (12, 16).
- FcγR-humanised animal models: Development of mice expressing human FcγRIIa (H131/R131 alleles) and FcγRIIb for *in vivo* ADE mechanistic studies (14, 24).
- FIPV vaccine re-engineering: Systematic evaluation of ADE-safe mucosal FIPV vaccine platforms (intranasal LNP-mRNA, VLP) as direct translational templates (32–35).
- IgG subclass dynamics: Longitudinal characterisation of afucosylation windows (19) and IgG4 class switching (20, 21, 23) in relation to ADE susceptibility in prospective clinical cohorts.
- Fc engineering clinical evaluation: Clinical evaluation of LALAPG, GASDALIE, and LALA-YTE Fc-modified therapeutic mAbs to establish the therapeutic window between ADE risk and beneficial ADCC effector function (30, 31).
- Variant surveillance: Continuous monitoring of antibody cross-reactivity and neutralisation profiles against emerging SARS-CoV-2 variants (37, 38, 44, 45).
- Nanomedicine platform comparison: Head-to-head comparison of LNP-mRNA, VLP, and PLGA nanoparticle vaccines in ADE-capable macaque and FIPV models, correlating antibody glycosylation profiles and nAb:non-nAb ratios (39–41).

## 11 Conclusions

Antibody-dependent enhancement in coronaviruses is a mechanistically nuanced phenomenon operating across molecular, cellular, and comparative biological levels. The convergence of classical FcγR-mediated extrinsic ADE—best exemplified by the FIPV model (32–35)—and intrinsic ADE mediated by FcγRIIbSOCS-IFN-I suppression (4, 5) provides SARS-CoV-2 with multiple molecular interfaces through which the host antibody response may be co-opted to facilitate rather than restrict infection. The FcγR–ACE2 cooperative model, experimentally supported by Kuzmina et al. (9) and mechanistically reinforced by Wang et al. (8), represents a significant mechanistic advance, though further *in vivo* validation and cryo-ET structural characterisation (10, 11) remain important research priorities.

Comparative veterinary immunology, and FIPV in particular, provides an irreplaceable model for coronavirus ADE research—the only natural host-pathogen coronavirus system in which ADE has been both unambiguously demonstrated as central to fatal pathogenesis and evaluated in the context of vaccination-enhanced disease (32–35). The nuanced immunological dynamics of transient afucosylation (19) and IgG4 class switching (20, 21) after mRNA vaccination highlight specific population groups and time windows requiring ongoing surveillance. The convergence of Fc engineering (LALA, LALAPG, GASDALIE) (30, 31), antigen design (prefusion-stabilised RBD-focused nanoparticles) (39–41), and delivery platform selection (LNP-mRNA, mucosal chitosan, VLP) (42, 43) provides a rational toolkit for jointly mitigating ADE risk in next-generation coronavirus vaccines. The comprehensive reviews of Nakayama & Shioda (37) and Tao et al. (38) confirm that this field is maturing rapidly.

## References

[1] Halstead SB . Dengue antibody-dependent enhancement: knowns and unknowns. Microbiol Spectr. (2014) 2:AID-0022-2014. doi: 10.1128/microbiolspec.AID-0022-2014 26104444

[2] Katzelnick LC Gresh L Halloran ME Baptista LC Fussell S Solano S . Antibody-dependent enhancement of severe dengue disease in humans. Science. (2017) 358:929–32. doi: 10.1126/science.aan6836 29097492 PMC5858873

[3] Takada A Kawaoka Y . Antibody-dependent enhancement of viral infection: molecular mechanisms and *in vivo* implications. Rev Med Virol. (2003) 13:387–98. doi: 10.1002/rmv.405 14625886

[4] Wan Y Shang J Sun S Tai W Chen J Geng Q . Molecular mechanism for antibody-dependent enhancement of coronavirus entry. J Virol. (2020) 94:e02015-19. doi: 10.1128/JVI.02015-19 31826992 PMC7022351

[5] Liu L Wei Q Lin Q Fang J Wang H Kwok H . Anti-spike IgG causes severe acute lung injury by skewing macrophage responses during acute SARS-CoV infection. JCI Insight. (2019) 4:e123158. doi: 10.1172/jci.insight.123158 30830861 PMC6478436

[6] Hoffmann M Kleine-Weber H Schroeder S Krüger N Herrler T Erichsen S . SARS-CoV-2 cell entry depends on ACE2 and TMPRSS2 and is blocked by a clinically proven protease inhibitor. Cell. (2020) 181:271–80:e8. doi: 10.1016/j.cell.2020.02.052 32142651 PMC7102627

[7] Walls AC Park YJ Tortorici MA Wall A McGuire AT Veesler D . Structure, function, and antigenicity of the SARS-CoV-2 spike glycoprotein. Cell. (2020) 181:281–92:e6. doi: 10.1016/j.cell.2020.02.058 32155444 PMC7102599

[8] Wang Z Deng T Zhang Y Yang Y An S Chen P . ACE2 can act as the secondary receptor in the FcγRdependent ADE of SARS-CoV-2 infection. iScience. (2022) 25:105462. doi: 10.1016/j.isci.2022.105462 35005526 PMC8719361

[9] Kuzmina NA Periasamy S Kedarinath K Hernandez K Atyeo C Dennison SM . SARS-CoV-2 antibody-dependent enhancement of infection depends on antibody binding to both ACE2 and Fc receptors. JCI Insight. (2026) 11:e197773. doi: 10.1172/jci.insight.197773 41729079 PMC12956010

[10] Akıl C Xu J Shen J Zhang P . Unveiling the structural spectrum of SARS-CoV-2 fusion by in situ cryo-ET. Nat Commun. (2025) 16:5150. doi: 10.1038/s41467-025-60406-z 40461447 PMC12134289

[11] Ke Z Oton J Qu K Cortese M Zila V McKeane L . Structures and distributions of SARS-CoV-2 spike proteins on intact virions. Nature. (2020) 588:498–502. doi: 10.1038/s41586-020-2665-2 32805734 PMC7116492

[12] Kibria MG Eaton A Bhatt DL Bhatt S Spriggs M Weaver EA . Antibody-mediated SARS-CoV-2 entry in cultured cells. Front Immunol. (2023) 14:1095429. doi: 10.3389/fimmu.2023.1095429

[13] Wieczorek L Zarling JM Kayser V . Evaluation of antibody-dependent Fc-mediated viral entry versus neutralization in SARS-CoV-2 infection. Vaccines (Basel). (2022) 10:1541. doi: 10.3390/vaccines10091541 35711449 PMC9193970

[14] Nimmerjahn F Ravetch JV . Fcγ receptors as regulators of immune responses. Nat Rev Immunol. (2008) 8:34–47. doi: 10.1038/nri2206 18064051

[15] Bournazos S Wang TT Ravetch JV . The role and function of Fcγ receptors on myeloid cells. Microbiol Spectr. (2016) 4. doi: 10.1128/microbiolspec.MCHD-0045-2016 28087938 PMC5240797

[16] Matveeva O . SARS-CoV-2 infection of phagocytic immune cells and COVID-19 pathology. Antiviral Res. (2022) 208:105452. doi: 10.1016/j.antiviral.2022.105452 36532011 PMC9751203

[17] Larsen MD de Graaf EL Sonneveld ME Plomp HR Nouta J Hoepel W . Afucosylated IgG characterizes enveloped viral infections and correlates with COVID-19 severity. Science. (2021) 371:eabc8378. doi: 10.1126/science.abc8378 33361116 PMC7919849

[18] Ferrara C Grau S Jäger C Sondermann P Brünker P Waldhauer I . Unique carbohydrate–carbohydrate interactions are required for high affinity binding between FcγRIII and antibodies lacking core fucose. Proc Natl Acad Sci USA. (2011) 108:12669–74. doi: 10.1073/pnas.1108455108 21768335 PMC3150898

[19] Van Coillie J Pongracz T Rahmöller J Geyer CE Vriesde M Ligthart PC . The BNT162b2 mRNA SARS-CoV-2 vaccine induces transient afucosylated IgG1 in naive but not in antigen-experienced vaccinees. EBioMedicine. (2023) 86:104393. doi: 10.1016/j.ebiom.2022.104393 36529104 PMC9756879

[20] Irrgang P Gerling J Kocher K Lapuente D Steininger P Habenicht K . Class switch toward noninflammatory, spike-specific IgG4 antibodies after repeated SARS-CoV-2 mRNA vaccination. Sci Immunol. (2023) 8:eade2798. doi: 10.1126/sciimmunol.ade2798 36548397 PMC9847566

[21] Uversky VN Redwan EM Makis W Rubio-Casillas A . IgG4 antibodies induced by repeated vaccination may generate immune tolerance to the SARS-CoV-2 spike protein. Vaccines. (2023) 11:991. doi: 10.3390/vaccines11050991 37243095 PMC10222767

[22] Koneczny I . A new classification system for IgG4 autoantibodies. Front Immunol. (2018) 9:97. doi: 10.3389/fimmu.2018.00097 29483905 PMC5816565

[23] Pérez CM Muñiz-Diaz E Roura X Prat Arrojo I Nogues N Castellà A . Post-vaccination IgG4 and IgG2 class switch associates with increased risk of SARS-CoV-2 infections. J Infect. (2025). doi: 10.1016/j.jinf.2025.106430 40113142

[24] Pandey JP Namboodiri AM Nietert PJ . Immunoglobulin GM and FcγR genotypes interact to contribute to the magnitude of ADCC against SARS-CoV-2 S-transfected cells. Immunogenetics. (2025). doi: 10.1007/s00251-025-01385-9 40888900 PMC12401749

[25] Cheung CY Poon LL Ng IH Luk W Sia SF Wu MH . Cytokine responses in severe acute respiratory syndrome coronavirus-infected macrophages *in vitro*. J Virol. (2005) 79:7819–26. doi: 10.1128/JVI.79.12.7819-7826.2005 15919935 PMC1143636

[26] Yin W Xu Y Xu P Cao X Wu C Gu C . Structures of the Omicron spike and ACE2 complex reveal a new mechanism of receptor interaction. Science. (2022) 375:1048–52. doi: 10.1126/science.abn7760 35133176 PMC8939775

[27] Shang J Wan Y Luo C Ye G Geng Q Auerbach A . Cell entry mechanisms of SARS-CoV-2. Proc Natl Acad Sci USA. (2020) 117:11727–34. doi: 10.1073/pnas.2003138117 32376634 PMC7260975

[28] de Alwis R Chen S Gan ES Ooi EE . Impact of immune enhancement on COVID-19 polyclonal hyperimmune globulin therapy and vaccine development. EBioMedicine. (2020) 55:102768. doi: 10.1016/j.ebiom.2020.102768 32344202 PMC7161485

[29] Lin CD Li Y Zhang YB Liu ZY Mu X Gu C . Ceftazidime is a potential drug to inhibit SARS-CoV-2 infection *in vitro* by blocking spike protein–ACE2 interaction. Signal Transduct Target Ther. (2021) 6:198. doi: 10.1038/s41392-021-00619-y 34006835 PMC8129692

[30] Bournazos S Gazumyan A Seaman MS Nussenzweig MC Ravetch JV . Bispecific anti-HIV-1 antibodies with enhanced breadth and potency. Cell. (2016) 165:1609–20. doi: 10.1016/j.cell.2016.04.050 27315478 PMC4970321

[31] Hale G . Living in LALA land? Forty years of attenuating Fc effector functions. Immunol Rev. (2024) 328:422437. doi: 10.1111/imr.13379 39158044 PMC11659930

[32] Pedersen NC . An update on feline infectious peritonitis: diagnostics and therapeutics. Vet J. (2014) 201:133141. doi: 10.1016/j.tvjl.2014.04.016 24857253 PMC7110619

[33] Pedersen NC . A review of feline infectious peritonitis virus infection: 1963–2008. J Feline Med Surg. (2009) 11:225–58. doi: 10.1016/j.jfms.2008.09.008 19254859 PMC7129802

[34] Olsen CW Corapi WV Ngichabe CK Baines JD Scott FW . Monoclonal antibodies to the spike protein of feline infectious peritonitis virus mediate antibody-dependent enhancement of infection of feline macrophages. J Virol. (1992) 66:956–65. doi: 10.1128/JVI.66.2.956-965.1992 1309922 PMC240797

[35] Vennema H de Groot RJ Harbour DA Dalderup M Gruffydd-Jones T Horzinek MC . Early death after feline infectious peritonitis virus challenge due to recombinant vaccinia virus immunization. J Virol. (1990) 64:1407–9. doi: 10.1128/JVI.64.3.1407-1409.1990 2154621 PMC249267

[36] Murphy BG Perron M Murakami E Bauer K Park Y Eckstrand C . The nucleoside analog GS-441524 strongly inhibits feline infectious peritonitis (FIP) virus in tissue culture and experimental cat infection studies. Vet Microbiol. (2018) 219:226–33. doi: 10.1016/j.vetmic.2018.04.026 29778200 PMC7117434

[37] Nakayama EE Shioda T . SARS-CoV-2 related antibody-dependent enhancement phenomena *in vitro* and *in vivo*. Int J Mol Sci. (2023) 24:4879. doi: 10.3390/ijms24054879 37110438 PMC10145615

[38] Tao T Zhao T Yang Y Jiang W Wang L Chen L . Antibody-dependent enhancement of coronaviruses: mechanisms, challenges, and therapeutic implications. Front Immunol. (2025) 16:1545623. doi: 10.3389/fimmu.2025.1545623

[39] Keech C Albert G Cho I Robertson A Reed P Neal S . Phase 1-2 trial of a SARS-CoV-2 recombinant spike protein nanoparticle vaccine. N Engl J Med. (2020) 383:2320–32. doi: 10.1056/NEJMoa2026920 32877576 PMC7494251

[40] Pati R Shukla M Bhol NK Mishra SP Mohapatra S Sahu SK . Innovations and trends in SARS-CoV-2 vaccine design. NPJ Vaccines. (2023) 8:84. doi: 10.1038/s41541-023-00689-7 37271785

[41] Vogel AB Kanevsky I Che Y Swanson KA Muik A Vormehr M . BNT162b vaccines protect rhesus macaques from SARS-CoV-2. Nature. (2021) 592:283–9. doi: 10.1038/s41586-021-03275-y 33524990

[42] Polack FP Thomas SJ Kitchin N Absalon J Gurtman A Lockhart S . Safety and efficacy of the BNT162b2 mRNA COVID-19 vaccine. N Engl J Med. (2020) 383:2603–15. doi: 10.1056/NEJMoa2034577 33301246 PMC7745181

[43] Baden LR El Sahly HM Essink B Kotloff K Frey S Novak R . Efficacy and safety of the mRNA-1273 SARS-CoV-2 vaccine. N Engl J Med. (2021) 384:403–16. doi: 10.1056/NEJMoa2035389 33378609 PMC7787219

[44] Zohar T Alter G . Dissecting antibody-mediated protection against SARS-CoV-2. Nat Rev Immunol. (2020) 20:392–4. doi: 10.1038/s41577-020-0359-5 32514035 PMC7278217

[45] Corti D Purcell LA Snell G Veesler D . Tackling COVID-19 with neutralizing monoclonal antibodies. Cell. (2021) 184:3086–108. doi: 10.1016/j.cell.2021.05.005 34087172 PMC8152891

[46] Bolles M Deming D Long K Agnihothram S Whitmore A Ferris M . A double-inactivated severe acute respiratory syndrome coronavirus vaccine provides incomplete protection in mice and induces increased eosinophilic proinflammatory pulmonary response upon challenge. J Virol. (2011) 85:12201–15. doi: 10.1128/JVI.06048-11 21937658 PMC3209347

[47] Lee WS Wheatley AK Kent SJ DeKosky BJ . Antibody-dependent enhancement and SARS-CoV-2 vaccines and therapies. Nat Microbiol. (2020) 5:1185–91. doi: 10.1038/s41564-020-00789-5 32908214 PMC12103240

[48] Arvin AM Fink K Schmid MA Cathcart A Spreafico R Havenar-Daughton C . A perspective on potential antibody-dependent enhancement of SARS-CoV-2. Nature. (2020) 584:353–63. doi: 10.1038/s41586-020-2538-8 32659783

[49] Ricke DO . Two different antibody-dependent enhancement (ADE) risks for SARS-CoV-2 antibodies. Front Immunol. (2021) 12:443. doi: 10.3389/fimmu.2021.640093 33717193 PMC7943455

[50] Guzman MG Alvarez M Halstead SB . Secondary infection as a risk factor for dengue hemorrhagic fever/dengue shock syndrome: an historical perspective and role of antibody-dependent enhancement of infection. Arch Virol. (2013) 158:1445–59. doi: 10.1007/s00705-013-1645-3 23471635

